# Evaluation of birth companions perinatal and peer support provision in two prison settings in England: a mixed-methods study

**DOI:** 10.1108/IJPH-09-2021-0099

**Published:** 2022-02-02

**Authors:** Gill Thomson, Rose Mortimer, Michelle Baybutt, Karen Whittaker

**Affiliations:** School of Community Health and Midwifery, University of Central Lancashire, Preston, UK and Dalarna University, Falun, Sweden; Anna Freud Centre, London, UK; School of Community Health and Midwifery, University of Central Lancashire, Preston, UK; School of Nursing, University of Central Lancashire, Preston, UK

**Keywords:** Women’s health, Health in prison, Health promotion, Women prisoners, Human rights, Qualitative research

## Abstract

**Purpose:**

This paper reports on insights from an evaluation of Birth Companions (BC) (a UK-based charity) perinatal support in two prison settings in England. The initiative involved the provision of group and/or one-to-one perinatal support and training women prisoners as peer supporters.

**Design/methodology/approach:**

A mixed-methods study was undertaken that involved observations of support groups and peer support supervision sessions (n = 9); audio recorded interviews (n = 33) with prison and health-care staff, women in prison, peer supporters and BC staff; analysis of existing routinely collected data by BC and notes undertaken during regular meetings (n = 10) with the BC Project Manager. Thematic analysis was undertaken supported by MAXQDA qualitative data analysis software.

**Findings:**

BC provided instrumental/practical support, emotional support, information support, signposting to services and advocating for women to the prison concerning their perinatal needs and rights. Key themes revealed that support had an impact on the lives of perinatal women by creating a safe place characterised by meaningful interactions and women-centred approaches that facilitated access to wider care and support. The service made a difference by empowering women and providing added value for peer supporters, prison, health-care and BC staff. Key enablers and strategies for the care of perinatal women and the delivery of perinatal support are also detailed.

**Originality/value:**

Through longitudinal data and the involvement of a range of stakeholders, this study evidences the subtleties of support provided by BC and the potential it has to make a difference to perinatal women in prison and those volunteering or working within the prison system.

## Background

Perinatal women in custody, which includes those who are pregnant or who have experienced a perinatal loss – miscarriage, termination, stillbirth, child removed following the birth – are a particularly vulnerable population with specific reproductive and psychosocial health needs (Home Office, 2007). Approximately 600 pregnant women enter prison in England each year, and about 100 babies are born in prison in the UK annually (Epstein and Brown, 2020), with complaints about a lack of readily available and rigorous data in this area being highlighted (Davies, 2019). Many pregnant women who are incarcerated often continue to engage in risky health behaviours such as smoking and substance misuse (Fowler and Rossiter, 2017). Currently, very few studies have been conducted examining the mental health experiences of pregnant women in prison in the UK. However, a small body of international research suggests that pregnant incarcerated women are likely to experience depression and anxiety, which (together with poor health behaviours) places them at high risk of adverse maternal and fetal outcomes (Mukherjee *et al.*, 2014; Shaw *et al.*, 2015). An Australian retrospective cohort study found that babies born to incarcerated women were more likely to be born premature, have low birth weight and require hospitalisation compared to community controls (Walker *et al.*, 2014). Pregnancy, neonatal loss and removal of a child following the birth are also associated with profound negative impacts on maternal mental health (Cacciatore, 2013; Eloff and Moen, 2003; Huang *et al.*, 2012), with women reported to display “deep grief” (Wood, 2008).

There are several international standards concerning the rights of women in prison, some of which relate to pregnant women. Rule 48 of the Bangkok Rules (United Nations General Assembly, 2010) states: “programmes for birthing companions, where they are available in the community, should also be made accessible to women in prison.” The United Nations Standard Minimum Rules for the Treatment of Prisoners (the Nelson Mandela Rules) (United Nations General Assembly, 2015) refers to pregnant women in Rule 28: “In women’s prisons, there shall be special accommodation for all necessary prenatal and postnatal care and treatment. Arrangements shall be made wherever practicable for children to be born in a hospital outside the prison. If a child is born in prison, this fact shall not be mentioned in the birth certificate.” Indeed, since 1957 international standards have prescribed that women prisoners should give birth in ordinary hospitals (Council of Europe, 2006). In England and Wales, it is stated that pregnant women should have the same key maternity entitlements to National Health Service (NHS) care as the general maternity population (Public Health England, 2018). Public Health England (2018) recommends a comprehensive range of provisions for pregnant women and new mothers in prison, including antenatal education and specialised peer support; there is some evidence that when enhanced perinatal care is provided, women can experience good outcomes, including decreased likelihood of preterm delivery and reduced recidivism (Bard *et al.*, 2016). However, many prisons in England and Wales do not provide the recommended services. Pregnant women in prison can face barriers in accessing adequate ante/postnatal care, including essential items, and their needs can be overlooked in prisons designed to house men (Walker *et al.*, 2014; Abbott, 2019, 2020). Prison staff are often unaware of the basic needs of perinatal women as well as Prison Service Instructions (PSIs) (National Offender Management Service (NOMS), 2014). Pregnancy-related PSIs concern the need to provide pregnant prisoners with adequate nutrition and rest, to support applications to Mother and Baby Units, and include guidance against transporting pregnant women in cellular vans or using handcuffs after arrival at hospital (NOMS, 2014).

Mother and Baby Units (MBUs) were introduced in UK prisons in the 1960s; in England and Wales, there are 12 women’s prisons, six of which have MBUs. MBUs are units that house mother and infant dyads (generally up to 18 months of age) and offer opportunities for mothers to bond with their baby in a more stable environment (O’Keefe and Dixon, 2015). Women in prisons without MBUs and who give birth in custody either gain places at MBUs at other prisons or are separated from their babies and go back into the main prison population. Similarly, women who give birth in prisons with MBUs can also be separated from their infants due to their perceived level of parenting capability and risk. Pregnant women in prisons without MBUs are unable to benefit from experience and expertise built up by staff in prisons that have MBUs, and non-MBU prisons face challenges in facilitating women’s applications for MBU places due to a lack of understanding (Sikand, 2015).

Currently, there are few published reports on perinatal interventions delivered in prisons (Ferszt and Erickson-Owens, 2008; Hogg, 2014; Shlafer *et al.*, 2018). The Minnesota Prison Doula project was a 12-week group-based support programme that provided pregnancy, birth and parenting services to incarcerated women in the USA (Shlafer *et al.*, 2018). Women who accessed the group reported more parenting confidence and more support from both other detainees and prison staff (Shlafer *et al.*, 2018). The UK-based nine session antenatal programme developed for vulnerable groups and delivered in community and prison settings was “Baby Steps” (Hogg, 2014). Parents who accessed the course felt that it improved their communication skills and appreciated the dedicated time and space to address and share their concerns. However, a key barrier for women prisoners was how a lack of control over their lives meant that they could not implement what they had learnt (Hogg, 2014). This highlights that programme content needs to be suitably adapted for the prison environment and prisoners’ specific needs. One of the only randomised controlled trials to examine an intervention designed for mothers and infants in the UK prisons (Sleed *et al.*, 2013) found that mothers in the New Beginnings programme remained stable on measures of parenting (parental reflective functioning and parent-child interaction) whereas mothers in the control group deteriorated in their parenting over time. This may suggest that interventions for parents in prison can ameliorate some of the negative impacts of prison on mother-infant interactions and bonding.

Birth Companions (BC) is a UK-based charity founded in 1996. The charity produced a Birth Charter (Birth Companions, 2016) and associated toolkit (Birth Companions, 2019) detailing recommendations for the care of pregnant women and new mothers in prison. Over the past decade, BC have been delivering antenatal/early parenting courses in various prisons in England that have a MBU. In 2016, BC developed a peer support programme with women prisoners trained to provide support to other perinatal women in custody. In May 2018, BC received funding from Her Majesty’s Prison and Probation Service (HMPPS) to provide perinatal support within two prison settings that do not have a MBU. The allocated funds were to deliver an adapted perinatal group programme (for pregnant women and those who have experienced a perinatal loss within the past 24 months), one-to-one support for perinatal women as an alternative or in addition to group support, recruiting and training women prisoners to become peer supporters and to undertake an evaluation. In this paper, we report on findings from the evaluation concerning how the BC activities were implemented and the impact of this support on women, peer supporters and staff (prison-related and BC). This work offers important insights into how perinatal women should be supported in prison, as well as how to optimise perinatal service delivery in the UK prison environment.

## Methods

### Design

This was an exploratory mixed-methods study, comprising observations, interviews and routinely collected sociodemographic data of women accessing BC services. The evaluation aimed to:

- provide an overview of BC’s activities in prison, including a snapshot of the women accessing these services;
- explore how BC implemented the perinatal support and different stakeholders’ perspectives on the value and impact of these services; and
- identify enablers to service delivery.

### Study context

Below we provide an overview of the two prisons and the perinatal provision provided at the time of the evaluation:

*Prison A* is an all-female closed establishment. All residents have a Personal Officer and an Offender Supervisor. Perinatal women – pregnant women and those who have had a live birth, miscarriage or termination within the past 12 months – are assigned a Family Engagement Coordinator, and there is a weekly multi-disciplinary team (MDT) meeting to discuss the care and needs of perinatal women. Prison staff do not receive specific training about the care of perinatal women. A midwife works 30 hours (four days) a week in the prison.

*Prison B* is an all-female closed establishment. There is a health-care centre with three inpatient beds and a family bonding unit to improve and strengthen family relationships. Every pregnant woman should receive a named Mother and Baby Officer, whose main responsibility is to support MBU applications. Additional duties include providing pregnancy-related items (such as maternity clothing) and organising and arranging a suitable birthing partner. A midwife provides support (e.g. antenatal clinic) one day a week in prison.

### Data collection and recruitment

Details of the different data collection activities undertaken at each of the two prisons are detailed as follows:

#### Interviews (group or individual).

Interviews were undertaken with BC staff (core staff and volunteers), prison and health-care staff, women in prison and peer supporters.

BC, prison and health-care staff were provided with (in person or by email) an information sheet, consent form and contact form (prison/ health-care staff only) and asked to complete and return the consent and contact form (via a pre-paid envelope or by email) to the evaluation team if they were willing to take part in an interview. A suitable time and date for a telephone interview was then organised. Suitable prison/health-care staff were identified by BC and key prison leads as those who were involved in the care and support of perinatal women.

Women/peer supporters were provided with a verbal overview of the study by BC staff and asked to complete a consent form if they were willing to participate in an interview and to indicate preference for a group or individual interview. Those who agreed were interviewed (individually or collectively as appropriate) when the researcher next attended the prison.

While different interview guides were devised for the different population groups (BC, prison/health-care staff and women/peer supporters), this was generally to capture variations in how the questions should be framed – all the interview guides were designed to answer the aims of the evaluation by exploring participants’ experiences and perceptions of BC services and views about facilitators and barriers to effective service delivery. All interviews were audio-recorded and transcribed in full for analysis purposes.

All the BC staff and prison/health-care staff were also invited to take part in a second interview (∼6 months after the initial interview) to capture their views over time.

#### Observations.

Observations were undertaken of perinatal groups and peer supporters’ supervision sessions with field notes written up after each observation. BC staff informed women and peer supporters (generally a week in advance of the researcher visit at the prison) that a researcher would be attending to observe the group (perinatal or supervision session). Women/peer supporters were advised that the observations were to gain an understanding of BC support and not to record any identifying or personal information. Verbal consent was sought from all women/peer supporters at the start of the observation. During the observation, the researcher sat among the women in the group and made anonymised notes in a non-obtrusive manner to avoid interrupting the flow of the discussions.

#### Routinely collected data.

BC routinely collects basic sociodemographic data (e.g. age, ethnicity, perinatal status) from all the women they support (where possible), and BC issued evaluation forms on an infrequent basis to collect women’s views of the support received. Any sociodemographic data and evaluation forms completed during the evaluation period were collected and reported.

#### Project lead meetings.

Regular meetings (and accompanying notes) were held with the BC Project Manager throughout the evaluation period to record decisions regarding the planning, implementation and delivery of support.

### Data analysis

All qualitative data, including interviews, fieldnotes, meeting notes and women’s written responses to BC evaluation forms, were uploaded into MAXQDA qualitative data analysis software. The data were analysed collectively to answer the aims of the evaluation, and to identify core issues across the data set; however, we were also mindful to identify and report on any variations by site as appropriate. Thematic analysis was undertaken using the approach developed by Braun and Clarke (2006). This involved an iterative process of close reading, identifying key codes, grouping codes into sub-themes and finally creating themes that were reflective of the whole data set. Two authors (GT and RM) were involved in the analysis process, with final analysis and interpretations agreed by all authors.

### Access and ethics approval

Ethical and governance approval was granted by the HMPPS National Research Committee (2018–331), and the University of Central Lancashire (STEMH 967). All research staff were fully HMPPS vetted to attend the prison and undertake research activities. Women prisoners are a vulnerable population for many reasons, and this vulnerability was considered during the interviews. Difficult topics were sensitivity addressed and it was detailed within the information sheet that any concerns concerning safety of individuals would be discussed with appropriate prison/health-care staff. In the event, no such concerns arose, and all women seemed to value the opportunity to share their experiences in a confidential space.

### Evaluation team and reflexivity

The evaluation was undertaken by three female researchers with expertise in perinatal issues (GT, KW), peer support provision (GT, MB) and needs of women in prison (RM, MB). While all the researchers held positive attitudes about the premise of BC support, there was a critical commitment to uncover how the perinatal activities were being implemented and experienced and to capture positive as well as negative practice as reported. While GT had worked with two members of the BC team previously (on a separate and unrelated research study), none of the research team had a prior relationship with any other participants. Data analysis was carried out by members of the evaluation team who had done prison visits and also those that had not; this meant that data analysis was not overtly influenced by the subjective experience of meeting the person and conducting the interview (at least in relation to the face-to-face interviews).

## Findings

An overview of all the data collected for the evaluation is detailed in Table 1, with further details provided below.

Overall, 33 interviews (three groups; 30 individual) took place with 32 individuals (four women, seven peer supporters, nine BC staff [three core and six volunteers] and 12 prison/health-care staff) and 10 meetings/discussions were held with the BC Project Manager. Five participants (2 core BC staff and three prison/health-care staff) also took part in follow-up interviews. All women prisoners interviewed were White, identified as female and were between 19 and 50 years of age. One was pregnant with her first child, two were pregnant and had previous children and one was approximately nine months post-partum. All prison staff were involved in the care/support of perinatal women and health-care staff included midwives and specialists who provided mental health support. Eight observation visits were undertaken (four at each prison), which comprised observations at seven perinatal groups and two peer support supervision sessions.

In the following sections, we present the data in three key sections (and in line with the aims of the evaluation). The first section “Overview of Birth Companions activities” provides details of the ethos and types of support that BC provided and includes a snapshot of the women accessing these services. The second section “Implementing and receiving Birth Companions support” explores how BC implemented the perinatal support and different stakeholders’ perspectives on the value and impact of these services. In the final section – “Enablers for perinatal support” – we describe enablers and strategies for how to improve the care of perinatal women and the delivery of perinatal support. Participant quotes include an identifier (PN–perinatal woman; PS-peer supporter; BCS–core BC staff member; BCV–BC volunteer; P/HCS prison/health-care staff), participant number and prison setting (as appropriate).

### Section one: overview of birth companions activities

BC operates within a trauma-informed, woman-centred and human rights framework, based on the recognition that women’s histories impact their current situation, behaviours and experiences. BC aims to ensure that women’s choices, privacy and dignity are respected, and that support is tailored to the needs of individual women. Their remit includes highlighting and rectifying instances of inappropriate care, particularly when it breaches PSIs (such as the lack of or inadequate support for MBU applications). This involves educating prison and health-care staff and coordinating meetings with senior staff to discuss prison service contraventions. An overview of the perinatal activities and support provided at each prison is detailed as follows:

#### Perinatal groups/support.

BC staff attended each prison on a fortnightly basis to deliver a perinatal group (to include antenatal/early parenting issues) and offer one-to-one support as needed. An eight-session rolling programme was devised (see Table 2). As BC’s remit was to support all perinatal women – those who were pregnant, had experienced a pregnancy loss or had a child removed from their care – the sessions were flexibly determined, taking into account the needs of those in attendance and avoided insensitive discussions.

Perinatal women who required individual support due to, for example, the sensitivity of their personal issue(s), group dynamics or complexity of needs were offered one-to-one support. In both prisons, the group was held in one large room, with individual support either taking place in a discrete area in the same space or in a separate area of the prison after the group had finished.

#### Peer supporters.

The peers’ remit was to support other prisoners by offering knowledge about the prison systems, accompanying women to meetings (where appropriate), offering reassurance and emotional support and to be a positive role model. All peers received monthly supervision from BC. Prison leads identified suitable women who: demonstrated empathy, a commitment to supporting perinatal women, were able to maintain boundaries and confidentiality, had good communication skills and had “suitable” offending histories. Pre-selected women were approached and asked to complete a BC application form if interested. Seven women were appointed at Prison B (two were later withdrawn due to unsuitable offences, one was released and one left the position) and four at Prison A. Training was delivered by BC over four days (or two days if the peers had prior “peer support” experience) and covered listening skills, being non-judgemental, how to support a woman following separation from her infant, understanding drug usage and women’s entitlements in prison.

Over the evaluation period (July 2018–March 2020), a total of 71 women actively engaged with BC support (20 – Prison A; 51 – Prison B) and 64 perinatal groups were provided (26 – Prison A; 38 – Prison B). Overall, 54 of the 71 women supported provided consent to share their sociodemographic details with BC. These data highlight that women who received support were more likely to be White British (77.8%), aged 22–34 years (64.8%), not married (66.7%) and pregnant (85.2%). The total number of eligible perinatal women was not available/collected by the prisons. An overview of the types of support provided (gathered during observations, discussions with BC Project Manager and by interviews across all participant groups) are detailed in Table 3.

### Section two: implementing and receiving birth companions support

Here we describe the ways in which BC activities shaped the experiences of women in prison and discuss the values of these services for women, peer supporters, BC and prison/health-care staff. The findings are organised around two key themes and associated sub-themes of “creating a safe space” and “making a difference.”

#### Creating a safe space.

We describe three sub-themes that detail how BC created a safe woman-centred space for women to benefit from embodied experiences of pregnancy and motherhood, and to receive the care and support they need.

### Enabling meaningful connections

It is important to highlight that there were challenges in identifying and referring eligible women to BC support at both prisons. These included delayed referrals, prison systems not recording women who had a perinatal loss, the degree of flexibility and autonomy peer supporters had when approaching women and women not receiving a “movement slip” to access prison activities, including the perinatal group. All these situations meant that there were avoidable delays in women receiving perinatal care. Despite these challenges, and once women’s access was enabled, the continual presence of BC staff at the perinatal group offered a valuable source of continuity in a complex prison system where “lot of things change” and where women “are not always informed” (BCV_3_Prison A):

Because of the nature, they [BC staff/volunteers and peer supporters] know their [women’s] history, they know what’s gone on, they’re working with these women all the time. So instead of having to tell somebody everything from the beginning they’re actually picking up with somebody and talking with someone they know, and they can talk through their problems with. (P/HCS_1_Prison A)

More importantly, within this space, the open, caring and non-judgemental approach that BC staff demonstrated was perceived to be a critical mechanism in forging positive and meaningful connections with women:

Very approachable, non-judgemental, and I think that’s what the women want. You know, they don’t want to be judged when they’re in prison and pregnant. They don’t want people looking down at them and BC don’t. They take them as they come, and deal with them appropriately. (P/HCS_1_Prison B)

Timely follow-up on woman-led requests for information or support was also considered crucial to maintain the BC-woman connections, as “if you don’t follow through what does that say? You know that you can’t trust us” (BCV_1_Prison A). It was considered essential to keep women notified if there were delays or difficulties in obtaining information or if there were any occasions when support could not be offered. This was particularly important in prison systems with lengthy delays and/or items being lost in the prison system (due to requiring security checks). As these women often came from complex backgrounds where “being let down” was common, it was important that BC kept women informed at all stages, offered what they said they would and did not “over promise to prevent disappointment” (BCS_1).

BC provided juice, fruit and snacks at the perinatal group as an incentive for women to attend. Some staff viewed this as less about the opportunity for a “treat,” and more about how receiving these items helped the women to feel valued:

[they] help them to feel at home and feel valued […] these people have brought something they want me to stay. They want to engage with me (P/HCS_4_Prison A)

Furthermore, while it was felt that the provision of “treats” could entice women to “see what the group was about” (P/HCS_4_Prison A) once they had attended, the perceived self-value became the underlying motivation for re-access. From a prison and health-care staff perspective, a key endorsement of the group’s success was that women continued to re-access; “the fact that they keep going, you know, for me says they get something out of it” (P/HCS_4_Prison B). One prison staff member reported that while women will sometimes refuse to attend professional appointments, this was not the case with the perinatal group:

So, when I’ve had this particular girl who hasn’t come to my appointments then I’ll meet up with the girls from the [BC] group and say “oh well how is she with you? Oh, she’s absolutely fine with us, yes comes to ours, engaged really well”, and you’ll see very conflicting behaviours. (P/HCS_3_Prison A)

This was also confirmed within women’s accounts, with women describing how they felt “welcomed,” “respected” and “accepted” and how the BC’s non-judgemental, “caring” and “genuine” approach was the key reason they continued to attend:

The Birth Companions women are so non-judgmental, they're just really nice people. They are welcoming and it makes you want to be there (PN1_Prison B)

### Providing woman-centred support

It was reported that not *all* women wanted to access the support, often due to highly sensitive situations (i.e. a recent infant separation) or complexity of life histories. For those who did, and in line with BC’s trauma-informed, woman-centred approach, the focus of the perinatal programme was “dependent on the needs of presenting women” (BCS_1). BC would request early information about attendant women from prison staff to help them prepare for their different needs, and topics that might be of relevance (or needed to be avoided), although in reality, this information was not always provided (at either prison). This was further compounded by encouraging “women having time and space to discuss whatever it is that’s on their mind” (P/HCS_4_Prison B). Being prepared and able to sensitively respond and engage with different women’s needs under these circumstances was considered one of the biggest challenges. The needs-led responsive approach to facilitate women-centred care, while introducing topics of relevance, was observed by staff:

It’s very much a protected space where they have, you know a topic each session but equally if one of the pregnant ladies comes with an issue that needs discussing and they need support with, then that overrides anything that the Birth Companions have got planned for that week (P/HCS_4_Prison B)

Some prison/health-care staff considered how a service tailored to women’s needs made women “feel a bit special”; with the woman-centred, safe space reported to have enabled women to feel “like a pregnant woman,” “a real person” (BCV_1_Prison A). The personalised nature of advice and extent to which, “Birth Companions put themselves out for you” (PN2_Prison B) in attempts to meet women’s needs, provided women with a sense of being cared for:

So knowing there is somebody somewhere who cares about you and is making sure you get your rights, even down to getting a bra that fits, and that is comfortable, something as simple as that is really, really important' (PS6_Prison B)

BC were able to offer invaluable practical items that the prisons “haven’t got the resources to provide” (P/HCS_1_Prison B). BC were also recognised as having benefits of being unrestrained by statutory working and having an “irreplaceable skill-set” (P/HCS_1_Prison A) of knowledge of the prison service, women’s rights and perinatal issues. This meant they were able to meaningfully engage women in a model of woman-centred care with sufficient dedicated time to provide essential support that perinatal women needed but often were unable to receive:

Because there's no staff to get information from, at least I know that there's Birth Companions to fall back on if you do have any questions – if you wait until Birth Companions come in, [you know] they will put your mind to rest (PN3_Prison A)

### Advocating for women and facilitating wider care

The virtue of creating a safe space for women and providing woman-centred care was believed to engender trust, which, in turn, made the women “more likely to talk” about their concerns (PS1_Prison A). Women were reported to be more willing to express their needs with BC staff when compared to interactions with other prison/health-care staff: “sometimes those ladies won’t open up to prison staff like they’re opening up to the Birth Companions” (P/HCS_1_Prison A). BC staff and peer supporters would work to empower women to share their issues with nominated prison staff, to raise awareness of their needs within the establishment and make follow-up possible. In other examples, there were occasions of BC staff and/or peer supporters accompanying women to chase up specific actions or supporting them in meetings on their behalf. One perinatal woman reported:

Birth Companions came to my meeting with Social Services to support me, and help me talk about the things I needed to say (PN1_Prison B)

An essential factor to implement BC support, as well as wider care for women, was via links with lead prison staff, though the relationships between BC and prison staff varied. At Prison B, the lead staff member was available and responsive, with other prison staff being instrumental in enabling BC service delivery; “it feels like we’re all part of a happy, jolly family” (BCS_2). An example of collaborative working included early resolution of a timetable clash to enable women’s access to the perinatal group. At Prison A, BC staff had to communicate almost exclusively with one key staff member, a situation that created difficulties when the named individual was unavailable. This was reported to “hinder our work, our ability to work effectively” (BCS_2). Prison A’s MDT initially offered the potential for BC to provide a “fuller” perspective of the women and to collaborate on needs-led care. However, a confidentiality breach (unrelated to BC staff) meant the format changed to a small core group, with other individuals (including BC) reporting for “five minutes or ten minutes” (BCS_1) on women under their care and then being asked to leave. While this new format was designed to protect confidentiality, BC and other health-care staff considered the revised MDT format to be a “disjointed” and “disruptive” custodial reporting platform, which presented a barrier to meaningful cross-case discussions (P/HCS_6_Prison A).

Despite the lack of consistent MDT practices and variable relationships with lead staff, BC would constantly strive to advocate for individual women and to improve wider prison practices. BC would report occasions of inappropriate use of restraints during the transportation of pregnant women and instances of prison officers staying in the room for medical consultations, scans and internal examinations without the women’s consent. When BC raised these issues, it was reported how prison staff were often “completely unaware” (BCS_1) of different rules for pregnant women. BC would also raise complaints of insensitive practices such as a woman being instructed (by a prison officer) to “cut up a sanitary towel” (BCS_2) in response to her request for breast pads and how the lack of food choice for pregnant women was leading to uneaten food and women going hungry. While there was no legal mandate for women to receive practical items (bras, firmer mattresses, additional pillows, etc.) BC would advocate for these items by presenting arguments as to how they would not only benefit the mother and her developing baby but also prison staff and practices; “such as a better mattress and maternity bra means that women will be more comfortable, sleep longer, and therefore less likely to self-harm or kick off” (BCS_2). Similarly, BC made the case that peer supporters can improve working practices for prison officers by responding to women’s requests and concerns. BC staff considered how presenting their arguments was a balance of managing and developing relationships with staff while making attempts to initiate positive change:

It's a bit of a tightrope because we do advocate for changes to the system and are quite openly critical in certain settings about what we see in prison and the failings that are there, so it's kind of maintaining the relationships individually with the key staff but equally feeling that we can influence things on a grounded platform as well. (BCS_2)

BC also advocated for and helped facilitate the introduction of the British Pregnancy Advisory Service (BPAS) charity in both prisons for women considering a termination post-16 weeks gestation. This service involved women being allowed to make a confidential call to a trained BPAS counsellor and BPAS then liaising with the prison to organise a termination as required.

While all pregnant women (and those recently separated from their infant) should receive information and support to make a MBU application, this was often not provided. While personal reasons could prevent women applying for a MBU, more commonly, women were in prison for substantial periods of time without being aware they could apply, and prison staff having “zero awareness” (BCS_1) of MBU eligibility criteria (i.e. a woman being incorrectly informed she could not apply to a MBU on remand). BC strove to ensure all eligible women were aware of their rights and were supported in the MBU applications process. Moreover, on occasion, women’s engagement with BC was considered to have contributed to their successful MBU application:

The input of Birth Companions and what they've done and having a very good social worker they have managed to turn things around, and we've had them going to the MBU. […] I don't think you'd have had that same outcome if you hadn't have had Birth Companions personally. (P/HCS_5_Prison B)

#### Making a difference.

In this section, we present two sub-themes that highlight the cognitive, physical, psychological and social benefits of BC support for perinatal women as well as salutary impacts for prison, and health-care staff, BC and peer supporters.

### Empowering women

There were numerous ways in which BC support served to empower women. In addition to the core activities of requesting and sourcing practical items for women, there were examples of innovative and proactive practice to advocate for women’s needs to be met. Examples included peer supporters speaking to core prison staff to coordinate a chapel service for a woman who had a late miscarriage and organising a gym class designed for pregnant women, thereby helping to improve women’s physical well-being:

One of the ladies I met wasn't 'gym fit' so she couldn't get to the gym, she can't get around to see the people that she needs to get that fixed, but I can. So, within half an hour she was 'gym fit', it was done' (PS6_Prison B)

Women expressed how the perinatal group and support from BC was the only forum which enabled them to receive reliable and comprehensive information and which helped them to feel better informed and more in control of their choices:

Writing the birth plans was really good, it helped me to learn, because there's lots of things I didn't know about pregnancy and birth. Like I know the words, but I don't know what they mean. But I've learnt about clamping the baby's cord and about how you can have a water birth, I thought you had to pay to have the water birth, but she told me it's free, so that's good. (PN1_Prison B)

Women valued the communal and safe space of the perinatal groups where they could share the expected or lived realities of pregnancy and motherhood:

I like giving that information out to someone else, the more women that breastfeed the better, you really don't realise how good it is for the children and how much time and money it saves for yourself, I tell everyone that (PN4_Prison A)

Furthermore, while group dynamics were not always optimum, positive group interactions were recorded within fieldnotes and reported by women, peer supporters and prison/health-care staff. The opportunities for perinatal woman to come together in a confidential, non-judgemental space enabled women to form friendships and bonds:

So even though all three were very different and very different life experiences, didn’t particularly know each other but they were all very supportive of one another. There was that common bond. (P/HCS_4_Prison B)

Importantly, group attendance made women feel less afraid, isolated and alone, as they could meet other perinatal women who they might not otherwise have come in to contact with:

I would have thought I'm the only pregnant person in the jail. That really helps make me calmer because prison is really scary (PN1_Prison B)

The perinatal group provided women with a positive sense of community – a rare experience in a prison environment:

Despite my own choices in ending my pregnancy I have witnessed what the group is capable of and it really is a group I look forward to – to build your confidence and feel a part of something special and mutual. I didn't feel alone anymore. (Woman#1, feedback form)

### Added value

Peer supporters, prison, health-care and BC staff all referred to how their engagement with the perinatal service had provided personal benefits. Some of the peer supporters expressed a personal sense of reward through knowing they had made a small difference in a woman’s life:

I do feel, if we've made the slightest difference, even so much as “right ok we will scrounge together some cereal for her” and the smile will be all over her face, and that tiny little thing which means nothing to us […] it's a big thing to her and it means something. And you feel as if you've done something rather than nothing. (PS1_Prison A)

The peer supporters also valued their involvement with BC as it demonstrated responsibility and positive rehabilitation:

They’ve [Birth Companions] said that they can write me a reference when I leave, so that’s helpful. […] it’s good to be able to show that you’re a Birth Companion (PS5_Prison B)

Some prison/health-care staff considered how BC involvement had led to prison staff and processes to be more accepting of providing woman-centred support: “I think it’s a lot better, there seems to be a lot more communication [about perinatal women]” (P/HCS_2_Prison A). Other prison staff reported that collaboration with BC meant that they had “a better understanding of women’s realities,” which on occasion helped them to “have a better relationship” with women (P/HCS_3_Prison A).

BC staff also considered how opportunities to provide intense support had important transferable learning opportunities of “how to work with them and how also how not to work with them” (BCS_2). The BC volunteers also reflected on how their role was “one of the most rewarding things I’ve ever done” (BCV_3_Prison B) and a “joy to be part of their life” (BCV_2_Prison A).

### Section three: enablers to perinatal support

In this final section, we report on key enablers for the care of perinatal women in general and the delivery of perinatal support. These findings are presented in Table 4, which summarises key enablers, the rationale and associated strategies to facilitate service delivery and to help improve outcomes.

## Discussion

In this paper, we report key insights from an evaluation of Birth Companions (BC) perinatal and peer support provision in two prison settings in England. The findings describe how BC created a safe space for care via the development of meaningful connections, providing woman-centred support and facilitating women’s access to wider care and support through advocacy and signposting. We also describe how BC support was felt to make a difference to women via cognitive, psychological and social benefits and the wider salutary value of BC provision to peer supporters, BC and prison/health-care staff. Finally, we detail enablers and associated strategies that may be of practical value when designing and organising services which seek to improve the care of perinatal women in prison. This work offers a distinct model of good practice for scale-up across the UK that should be supported by prison and health-care systems. Many women in prison are mothers of infants or young children and are considered an “invisible” group due to their needs often being unmet (Kincaid *et al.*, 2019). Research concerning interventions with incarcerated perinatal women is of critical importance and represents an important opportunity to improve public health (Shaw *et al.*, 2015).

Like other interventions designed for highly vulnerable perinatal women, both in the community and in prison, we found that access to targeted perinatal support helped to improve women’s confidence, their ability to communicate their needs and opportunities for positive social connections (Balaam and Thomson, 2018; Hogg, 2014; Shlafer *et al.*, 2018; Thomson *et al.*, 2017). An important facet of BC work was that it was delivered over an extended period through fortnightly prison visits. This consistency and reliability facilitated trust between women and BC staff and resulted in meaningful relationships. BC staff worked hard to advocate for women and sought to ensure that women’s rights, entitlements and wider needs were recognised and met by the prison where possible.

While there are no existing studies of perinatal peer support in prison, our findings reflect wider research which has shown that women value the reassurance, information and social networks of peer support provision (Trickey *et al.*, 2018; Thomson and Crossland, 2019). Furthermore, the findings that BC provision had benefits to peers and staff resonate with insights as to how third-sector organisations can positively influence statutory provision (Aiken and Thomson, 2013) and peer supporters reap benefits from increased self-esteem, self-confidence and personal growth (Fletcher and Batty, 2012; Schwartz *et al.*, 2003).

This work has helped to identify specific strategies to improve the care and support of perinatal women, as well as how to optimise the delivery of perinatal support. A number of these recommendations align with the UK *Better Birth* maternity transformation programme (NHS England, 2015) agenda and the NHS Long-Term Plan (NHS England, 2019), including the need to maximise continuity of care and opportunities for trust and relationship-building between staff and women (such as via weekly, rather than fortnightly contacts and increased flexibility for peer contacts). Further, there is a need to develop systems for information sharing and interdisciplinary working to ensure needs-led care for women. Critically, if BC are to be maximally effective, they must be supported by accommodating prison systems that ensure timely needs assessment, information sharing and communication, and trust and respect between prison staff, BC staff and peer supporters.

It is recognised that prison staff working in prisons without a MBU often lack experience and expertise of women’s perinatal needs and further training for prison staff is required. Training for prison staff concerning how traumatic experiences impact negatively in the perinatal period is needed (Seng and Taylor, 2015). Another key training need relates to MBU applications. During 2018–2019 (over the period of the evaluation), 60 women moved into a MBU in England and Wales and while 75% of MBU applications were successful, MBU spaces are underused as some eligible women do not apply (Ministry of Justice, 2020). This means that there are lost opportunities to prevent unnecessary separations and to enhance maternal-infant relationships (Sikand, 2015). As pregnancy offers an important impetus for positive change (Hall and van Teijlingen, 2006), opportunities to facilitate and optimise this change, such as via MBU placements and peer support, are essential. However, this work also requires nominated prison staff with dedicated and protected responsibilities, and who have the passion and commitment to support perinatal women.

A further area to highlight relates to the need for MDT working. MDTs offer many benefits including bringing together expertise and skills to assess, plan and coordinate care, particularly for individuals with complex care needs (Aikman, 2018; Radcliffe *et al.*, 2020). Many women in prison have experienced multiple disadvantages due to an array of complex life experiences, including sexual exploitation, childhood sexual abuse, domestic violence, mental health problems and substance misuse needs. Pregnancy or perinatal loss, combined with prison add further layers of vulnerability (Fowler and Rossiter, 2017). Regular MDTs should be established that include all key agencies, with suitable confidentiality agreements in place, to facilitate collaboration between different professionals, maximise offers of woman-centred care and improve outcomes (Aikman, 2018; Radcliffe *et al.*, 2020).

*Strengths and limitations:* The strengths of this work relate to capturing insights from different stakeholders, therefore enabling a rich picture informed by multiple perspectives. The longitudinal nature of the evaluation also meant that insights into the challenges and remedial actions could be captured over time. While the number of interviews with imprisoned women were relatively low, this was often due to women having just received bad news or not feeling well on the day of the visit. The addition of observational visits meant that women’s views and experiences were included even when they chose not to participate in an interview. Overall, we were able to capture valuable insights into the challenges and facilitators to perinatal support in prison settings, as well as describing the value of BC work and the potential positive impacts for women and others. Future work should continue to evaluate perinatal interventions in prisons through a combination of in-depth qualitative research, as well as trials to examine the outcomes of specific interventions to ensure that the best quality, joined-up care can be provided.

## Conclusion

Pregnant women in prison are a highly vulnerable group; many have experienced chaotic lives and multiple disadvantages. Prison represents an important opportunity to meet the needs of these women. This paper reports on insights from an evaluation of perinatal support provided by Birth Companions, a UK-based national charity – within two female prisons in England. Findings describe how Birth Companions created a safe space for perinatal women, characterised by meaningful connections, women-centred approaches, advocacy and facilitation to wider care. The service was reported to make a difference by empowering women, as well as offering benefits to prison/health-care staff, peer supporters as well as as well as Birth Companions staff. Key enablers to improve the care of perinatal women and the delivery of perinatal support are detailed. These findings should inform the development of perinatal interventions in prison environments and guide prisons and third sector organisations towards working together effectively, to facilitate the best possible outcomes for women and infants.

## Figures and Tables

**Table 1:** Overview of data collection

Participants	No. of participants	No./types of data collection methods	No. interviewed in each prison setting
Perinatal women	4	Four individual face-to-face interviews	Two in Prison A Two in Prison B
Peer supporters	7	Two group face to face interviews One individual face to face interview	Four in Prison A Three in Prison B
Birth Companions core staff*	3	One group face to face interview Four individual telephone interviews	Staff worked over both prisons
Birth Companions volunteers/staff	6	Six telephone interviews	Three in Prison A Three in Prison B
Project meetings	10	10 telephone discussions	
Fieldnotes	9	Observations of eight perinatal groups and two peer support supervision sessions	Four perinatal groups in Prison A; three perinatal groups in Prison B One peer support supervision in Prison A ; one peer support supervision in Prison B
Prison staff*	8	Nine telephone interviews	Three in Prison A Four in Prison B One interview with a strategic lead
Health-care staff*	4	Six telephone interviews	Three in Prison A One in Prison B
Evaluation data	8	BC evaluation forms	One in Prison A Seven in Prison B

Note: *Some participants took part in multiple interviews

**Table 2:** Overview of topics for perinatal group

Session 1	Common pregnancy complications; making decisions for labour, birth and beyond
Session 2	Pelvic floor; pregnancy exercises and self-care
Session 3	Hormones during labour, birth and beyond; induction; third stage of labour
Session 4	Pain relief options
Session 5	Birth positions
Session 6	Infant feeding
Session 7	Birth planning
Session 8	Postnatal recovery (and new-born care)

**Table 3:** Overview of types of support provided during perinatal activities

Category of support	Examples
Instrumental/practical support	Ensuring women received essential items, such as pregnancy packs (e.g. additional food packs), pregnancy pillows, maternity bras, maternity clothes and breast pads. Providing parenting books, emergency phone credit (on release) and consulting with key agencies on women’s behalf
Emotional support	Providing opportunities for women to share frustrations, fears and concerns, e.g. losing custody of children, concerns over mental health, birth-related fears. Liaising with prison staff to enable women to receive a scan photo, organising a suitable birth partner, for photos/prints of the baby’s hands and feet to be taken post-birth, providing women with materials to make cards for their baby or keep a pregnancy journal and providing women with baby clothes (for the infant to wear and to be returned to the woman)
Informational support	Providing information on practical items women should receive (i.e. additional food), women’s rights during transfer to hospital and during procedures, child protection procedures and proceedings, MBU applications/placements, applying for re-categorisation and specialist information, e.g. applications to Home Office (immigration status)
Signposting	Signposting women to available services, e.g. Family Rights Group (for women with Social Services involvement), housing services, counselling services, mental health and health care
Advocacy	Advocating for women with the prison around their perinatal needs and following correct Prison Service guidelines (e.g. for MBU applications and maintaining dignity while women are being transported to/at hospital)

**Table 4:** Key enablers for perinatal care and support

Enabler	Rationale	Strategy
*Consistent and comprehensive systems to identify/refer eligible women*	▪ Challenges in identification of women were noted at both prisons (particularly for those who had a perinatal loss), leading to delays in timely support	▪ Regular (i.e. weekly) lists of *all* eligible women shared securely (including name), prison ID (for follow-up) and brief perinatal history ▪ Women’s perinatal history (in the past two years) recorded at time of admission on IT systems to maximise referrals
*Better training and education of prison staff*	▪ Evidence of variable levels of appreciation by prison staff of the physical, emotional and social needs of perinatal women	▪ All staff working in women’s prisons to receive training (mandatory, yearly updates) into the needs and entitlements of pregnant women and wider issues faced by perinatal women
*Ensuring all women are aware of/supported to complete MBU applications*	▪ Overall, there was a lack of knowledge and dedicated support for MBU applications	▪ A clear pathway with named (and trained) prison officers with protected time to distribute information (e.g. leaflets, verbally) and support MBU applications ▪ Other prison/ health-care staff to receive training in MBU applications
*Maximising opportunities to receive perinatal support*	▪ Women able to attend perinatal activities found them to be an important source of information and opportunities for practical, social and emotional support	▪ Funding provided to enable weekly (group and/or individual) perinatal support ▪ Prison staff to be informed of perinatal support and for “movement slips” organised in a timely fashion ▪ Peer supporters require flexibility to approach and support women ▪ Provision of robust and comprehensive referral pathways
*Collaborative multi-disciplinary practices*	▪ Collaborative multi-disciplinary practices can help ensure needs-led support ▪ While positive evidence of effective multi-disciplinary practice was observed, key gaps included a general lack of commitment, communication and awareness	▪ Dedicated prison staff (with at least one named deputy) with sufficient time and authority to action and monitor perinatal delivery ▪ Multiple and frequent opportunities to provide information of service delivery (such as BC support), e.g. within mandatory training, existing prison/staff meetings, email updates, prison radio, leaflets and posters ▪ Multi-disciplinary meetings including representatives from relevant prison, health-care and third sector organisations should be established
